# Fuzzle 2.0: Ligand Binding in Natural Protein Building Blocks

**DOI:** 10.3389/fmolb.2021.715972

**Published:** 2021-08-18

**Authors:** Noelia Ferruz, Florian Michel, Francisco Lobos, Steffen Schmidt, Birte Höcker

**Affiliations:** ^1^ Department of Biochemistry, University of Bayreuth, Bayreuth, Germany; ^2^ Computational Biochemistry, University of Bayreuth, Bayreuth, Germany

**Keywords:** web server, protein evolution, protein design, protein fragment, flavodoxin-like fold, periplasmic binding protein

## Abstract

Modern proteins have been shown to share evolutionary relationships *via* subdomain-sized fragments. The assembly of such fragments through duplication and recombination events led to the complex structures and functions we observe today. We previously implemented a pipeline that identified more than 1,000 of these fragments that are shared by different protein folds and developed a web interface to analyze and search for them. This resource named Fuzzle helps structural and evolutionary biologists to identify and analyze conserved parts of a protein but it also provides protein engineers with building blocks for example to design proteins by fragment combination. Here, we describe a new version of this web resource that was extended to include ligand information. This addition is a significant asset to the database since now protein fragments that bind specific ligands can be identified and analyzed. Often the mode of ligand binding is conserved in proteins thereby supporting a common evolutionary origin. The same can now be explored for subdomain-sized fragments within this database. This ligand binding information can also be used in protein engineering to graft binding pockets into other protein scaffolds or to transfer functional sites *via* recombination of a specific fragment. Fuzzle 2.0 is freely available at https://fuzzle.uni-bayreuth.de/2.0.

## Introduction

A main function of proteins is the binding of molecules such as other proteins or smaller compounds. For example, the entire machinery of metabolic pathways consists of proteins that bind various substrates and catalyze diverse reactions (Schmidt and Dandekar, 2002). Despite this apparent diversity proteins were often reused in the course of evolution and their reactions adapted to perform different functions. In fact, todays diverse set of proteins and their associated functions are the product of mutation, recombination and duplication events (Horowitz, 1945; Jensen, 1976; Ohta, 2000; Sikosek and Chan, 2014).

For a long time, protein domains have been considered as the evolutionary unit, being structurally discrete and independently folding. However, the analysis of the known sequence and structure space in recent years led to a renewed insight on an old concept: Modern proteins might have arisen from a set of primordial peptides to increasingly larger subdomain-sized fragments (Alva and Lupas, 2018; Romero-Romero et al., 2021). Based on sequence and structural similarities it is possible to infer likely evolutionary relationships of proteins, even of different folds (Farías-Rico et al., 2014). The examples provided by Farías-Rico et al. and Alva et al. show how nature used these ready-made pieces in the evolution of modern protein diversity.

A number of studies have now identified several subdomain-sized fragments as common evolutionary units (Alva et al., 2015; Nepomnyachiy et al., 2017; Ferruz et al., 2020). The database of subdomain-sized fragments that we developed previously is accessible *via* a web interface to allow individual analysis (Ferruz et al., 2020). These conserved fragments often participate in ligand binding, including nucleotides, nucleotide-derived cofactors, or metal ions (Bharat et al., 2008; Laurino et al., 2016; Goncearenco and Berezovsky, 2015; Romero Romero et al., 2018; Longo et al., 2020; Narunsky et al., 2020). This clearly indicates a key role of ligand interactions in the evolution of these ancestral building blocks.

To include this important aspect, we have updated Fuzzle to allow systematic searches for ligands and to enable a better understanding of the evolution of protein fragments. Fuzzle 2.0 enables the analysis of non-covalent interactions of protein-ligand complexes. Additionally, it now also allows searching for homologous fragments that nature has reused as building blocks that bind the same ligand. Here, we demonstrate its new capabilities using as an example a periplasmic binding protein (PBP). We show how PBPs contain a conserved fragment that is associated with several ligands and we highlight its homologous relationships to several other superfamilies. This conserved protein building block is examined from an evolutionary as well as a protein engineering perspective.

## Materials and Methods

### Database

The Fuzzle database uses SCOPe (Fox et al., 2014) to identify protein domains. SCOPe is a hierarchical database that sorts domains into folds, superfamilies and families. We first updated Fuzzle to include SCOPe release 2.07. Common sub-domain fragments were identified as previously described (Ferruz et al., 2020). In particular, we created hidden Markov model profiles for each domain in SCOP95 2.07 using the HH-suite (Söding, 2005). These domains were compared all-against-all using HHsearch and then structurally superimposed using TM-align (Zhang and Skolnick, 2005). TM-align calculates the RMSD based on Cα-atoms. The data is stored in the database as ‘SCOPe 2.07 PSI’. We then filtered hits (pairs of domains that have a fragment in common) from different folds, with an RMSD <3 Å, HHsearch probability over 70%, length between 10 and 200 amino acids and TM-score > 0.3. Hits were allowed to have sequence alignments at most 25% longer than the structural alignments. Since SCOPe lacks coordinates of bound ligands, we retrieved the coordinates from the original PDB entries using a 4 Å distance cutoff for any heavy atom. To stay consistent with the PDB definition, all ‘HETATM’ entries were considered as ligands, including modified residues. We added the corresponding ligands’ coordinates. In cases where a ligand is bound in between multiple domains, it will appear with all domains where it shows an interaction based on the cutoff.

### Website

The web interface contains several updates from its predecessor version. It is now possible to search for ligands in two ways: either by its PDB (three-letter) code (e.g., ATP for adenosine-5′-triphosphate) or by its SMILES (Weininger, 1988). SMILES searches in Fuzzle 2.0 not only find ligands that are identical, but users can also search ligands that are more than 70% similar. Similarity searches use topological RDKit fingerprints (default parameters: minimum path size: 1 bond - maximum path size: 7 bonds - fingerprint size: 2048 bits - number of bits set per hash: 2 - minimum fingerprint size: 64 bits - target on-bit density 0.0) with Tanimoto similarity coefficient (Godden et al., 2000). Moreover, SMILES searches allow to identify sub- or superstructures of a compound (e.g., adenosine and inorganic phosphate as substructures of ATP).

The database has now been extended to include additional information about ligands and fragments. For example, the fragment analysis page now contains a table that includes the statistics of the fragment: A representative domain that contains each fragment (selected as the domain with most network connections to other domains that also contain that fragment, such as domain ‘d1jw9b_’ for fragment 1: https://fuzzle.uni-bayreuth.de/2.0/fragments/network/fragment/1), the number of domains that contain the fragment, the average fragment length, involved folds, and the ligands bound to the fragment. In a detailed view it is possible to visualize protein-ligand interactions in the context of fragments using the NGL Viewer (Rose et al., 2018). To analyze the interactions, one can toggle different interaction types, compute distances, and show surface representations. The relationship tables between all SCOPe categories have been updated to reflect the ligand information. Tables and networks containing this updated information can be downloaded as CSV or JSON files. Superpositions of fragments are available as PyMOL sessions. Additionally, a fragment search was implemented to allow finding fragments depending on their ligands, SCOPe category or length. The web frontend was altered to reflect these changes. To this end, we use Django (version 1.11), PostgreSQL, and JavaScript. The style of the web site relies on the Bootstrap framework (version 4.0). Other software technologies used in Fuzzle 2.0 include JQuery (jquery.com), graph_tool (graph-tool.skewed.de), Datatables (datatables.net), and D3js (d3js.org) to visualize the data.

### Analysis of Ligands Binding the PBP-like Fragment

Ligands that are commonly known to be additives to crystallization screens or other experiments were excluded from our ligand-binding analysis to the PBP fragment. These additives are listed in BioLiP (Yang et al., 2013) and in this case correspond to: ACM, ACT, ACY, CIT, CL, EDO, FMT, GOL, MPD, MPO, MSE, NA, PEG, PO4, SO4, and TRS. CA and MSO were also removed from the set. Sequence alignments in the Supplementary Material were retrieved from each of the pairwise alignments to d2fn9a_ and grouped by superfamily. The webpage allows to filter out these crystallization artifacts and post-translational modifications with toggle buttons (e.g: https://fuzzle.uni-bayreuth.de/2.0/fragments/table/).

## Results

### New Fuzzle Features

The original Fuzzle database already contained a large number of conserved fragments that are shared between folds thereby illustrating a remarkable connectivity of the protein universe. The inclusion of the SCOPe 2.07 database increased the size of domains and thereby the number of pairwise hits (Supplementary Table S1). As with the previous version, fragment hits were clustered to incorporate the possibility of multiple distinct fragments being found within a single protein domain. If standard cutoffs are applied, we still observe the same power-law distribution of domain connectivity, with few domains accumulating most of the network's links in a highly populated major component (Ferruz et al., 2021).

A major improvement is the addition of ligand information (Supplementary Figure S1). It is now possible to search for ligands in two ways: either by its PDB code (e.g., adenosine-5′-triphosphate: ATP) or by its SMILES. SMILES searches not only provide identical or 70% similar ligands, but also superstructures or substructures of the compound using Tanimoto coefficients. To cite an example, searching for substructures of ATP would also provide all fragments that bind substructures of it, like adenosine or inorganic phosphate.

In addition, we also enable visualization of networks of proteins bound to certain ligands and provide this information in a downloadable table. The table includes not only the statistics of the fragment and the most connected entry as a representative but also all ligands that are bound to the fragment. It is possible to retrieve additional information for each ligand and to directly visualize the protein-ligand interactions in the context of the selected fragment using the NGL Viewer. One can toggle interactions like π–π stacking, hydrogen bonds, compute distances, and show surface representations.

### A PBP-like Conserved Fragment

**FIGURE 1:**
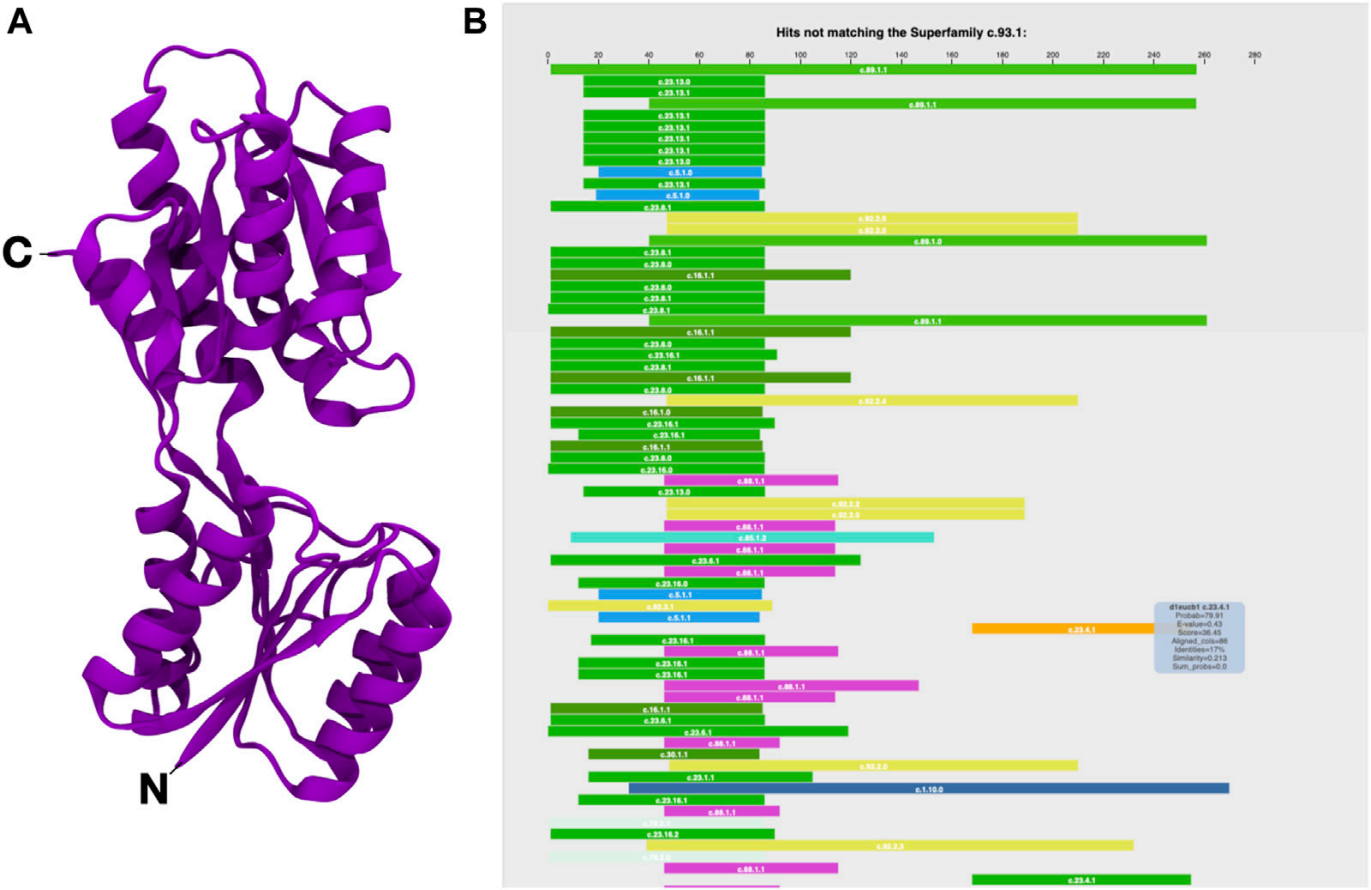
The ribose binding protein from *Thermotoga maritima* (tmRBP, d2fn9a_). **(A)** Structure (PDB 2FN9) and **(B)** HHsearch overview (https://fuzzle.uni-bayreuth.de/2.0/hh/hhs-raw/d2fn9a_/). The query sequence (d2fn9a_) is taken as reference for the alignment of the proteins found (residues 1–280). Each bar represents a hit that is related with the query domain but does not belong to the same superfamily. The position of the bar matches the location of the hit related to the query’s sequence. Colors differentiate superfamilies. Extra information on the hits can be found by mousing over. Note that in this summary diagram hits that were found to other proteins of the same superfamily are not shown.

Fuzzle 2.0’s new features enhance the analysis of fragments. Here we want to illustrate them by exploring the evolutionary relationship of a member of the periplasmic binding protein-like I fold (PBP-like I, c.93) (Figure 1A). In Fuzzle we also find hints of these evolutionary relationships between PBPs and other folds. Here, we use the ribose binding protein from *Thermotoga maritima* (tmRBP, d2fn9a_). It belongs to the PBP-like I superfamily c.93.1 (Cuneo et al., 2008). We observe that d2fn9a_ appears in an unusually high number of hits either as query or subject (altogether 2028) with other protein domains in the database without any cutoffs, as can be queried with the software Protlego (Ferruz et al., 2021). 1,566 of those hits correspond to local alignments shared with domains that belong to other superfamilies and folds. This domain is more connected than observed for the average domain in Fuzzle (172 hits/domain). If we focus on standard cutoffs, we obtain 121 hits, belonging to 15 superfamilies and 9 folds. These numbers indicate that domain d2fn9a_ shares several conserved fragments with other domains.

**FIGURE 2:**
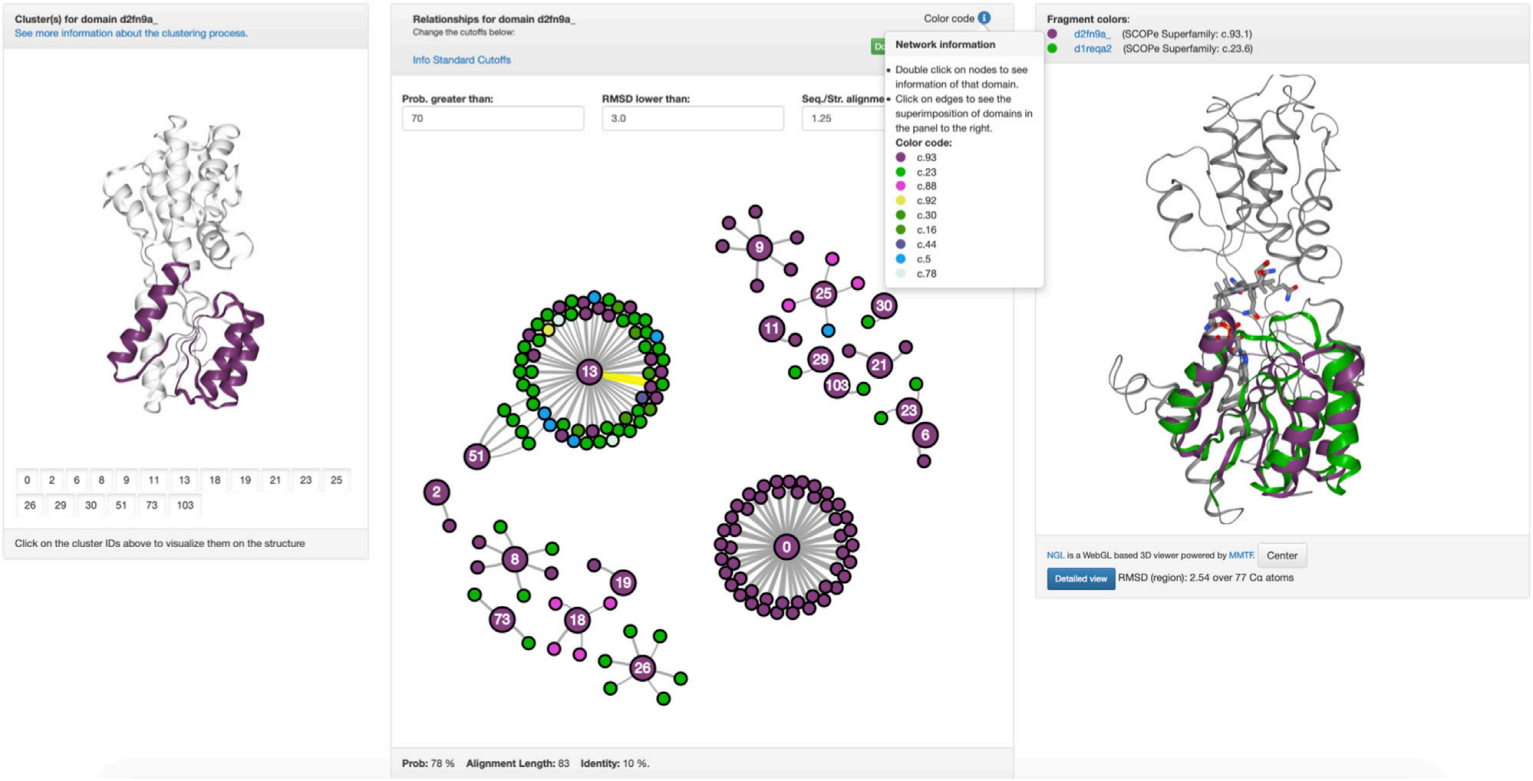
Conservation in tmRBP. (c.f. https://fuzzle.uni-bayreuth.de/2.0/hh/fragment_graph/d2fn9a_/). **Left:** The panel highlights each hit in d2fn9a_. In this case, cluster 13 has been selected, the main object of this study. **Middle:** The clusters of d2fn9a_ are shown, where each interactive node is a domain, linked to other domains when they share a fragment. Each ‘island-like’ cluster corresponds to a set of proteins that have a fragment in common. Nodes are colored according to their folds **(top)**. Mousing over an edge it gets highlighted (yellow) and the alignment parameters in the footer are shown **(bottom)**. An individual PyMOL session of superposed structures for each cluster can be downloaded **(top green button)**. **Right:** Upon clicking on an edge between two nodes **(middle panel)** the superposition of the structures with their fragment colored according to their fold will be shown on the right.

This high evolutionary connectivity can be viewed in Fuzzle when looking at the HHsearch hits to sequences of other superfamilies (Figure 1B). tmRBP shows an unusual number of local alignments in its N-terminal region, indicating a conserved fragment. We thus decided to look in detail at this fragment and characterize it. This is possible by using domain-centered networks in Fuzzle (Figure 2). In this representation, the domain in question is defined as an interactive circle, and other domains that have fragments in common are linked to it. d2fn9a_ always appears as the center of each ‘island’ or cluster. In this representation, we show all hits that surpass the previously described cutoffs but unlike Figure 1B it includes hits with domains from the same superfamily as well. The ID numbers (Figure 2, left) are not contiguous since not all fragments fulfill the user-defined cutoffs. To discern them from the previously defined fragments, we will call these connections within the domain-centered networks ‘clusters’. Clicking on the identifiers to the left depicts the cluster in the domain (Figure 2, left).

For tmRBP, Fuzzle identifies a total of 153 hits to 136 other domains (Figure 2, Supplementary Table S1) using the standard cutoffs. These hits can then be grouped into 18 clusters that map to different regions of tmRBP (Figure 2, Supplementary Table S2). The coloring scheme matches throughout Fuzzle 2.0 representing the individual folds. For example, sequences that are shown in green in Figure 1B also appear as green nodes in Figure 2. We can thus infer that these sequences have cluster 13 in common. The fragment position confirms this observation (positions 11–87, Supplementary Table S2, Figure 2). With 63 domains in this subgraph, cluster 13 constitutes d2fn9a_‘s most promiscuous fragment. Structurally, it contains the three N-terminal helices and four β-sheets, with the first β-sheet not necessarily present in all domains. In total, cluster 13 spans domains from 8 folds to 12 superfamilies: c.16.1 (Lumazine synthase): 4 domains, c.23.1 (CheY-like): 2 domains, c.23.13 (Type II 3-dehydroquinate dehydratases): 9 domains, c.23.16 (Class I glutamine amidotransferase-like): 9 domains, c.23.6 (Cobalamin (vitamin B_12_)-binding domain), c.23.8 (N^5^-CAIR mutase (Phosphoribosylaminoimidazole carboxylase, PurE): 2 domains, c.30.1 (PreATP-grasp domain): 11 domains, c.44.3 (PIWI domain N-terminal-like): 1 domain, c.5.1 (c.5.1: MurCD N-terminal domain): 3 domains, c.78.2 (Aspartate/glutamate racemase): 2 domains, c.92.3 (PrpR receptor domain-like): 1 domain, and c.93.1 (Periplasmic binding protein-like I): 16 domains.

### Ligand-Binding in the Conserved PBP-like Fragment

One of the major goals of Fuzzle 2.0 is not only to update our platform for evolutionary analyses but also to facilitate searches for suitable fragments to design protein chimeras. In the case of d2fn9a_, parts of the protein that correspond to cluster 13 could be replaced with a homologous and structurally well-superimposed fragment of another protein that possesses an interesting function (Ferruz et al., 2021). d2fn9a_‘s cluster 13 is a good candidate for this study, as it contains more than 63 direct hits in the same and other superfamilies that contain the same structural fragment with large deviations in sequence and function. These deviations, although large, are still remnants of a remote homologous ancestor, but more importantly, provide a wide range of functionalities that we can exploit for protein design purposes. In this example, we have looked at the ligand-binding capabilities of the 63 domains containing d2fn9a_‘s cluster 13. These fragments, provided there are no structural clashes, could hence be potential candidates for replacement as previously achieved experimentally with the PBP-flavodoxin-like chimera (PDB id: 4QWV). Besides, they could represent a starting point for protein engineering, especially those fragments that entirely encapsulate a ligand offering an opportunity for binding site transfer. This can for example be done with the recently published Protlego tool (Ferruz et al., 2021). Here, we have characterized the ligand-binding proteins containing cluster 13 and analyzed their prospects for protein engineering.

**FIGURE 3:**
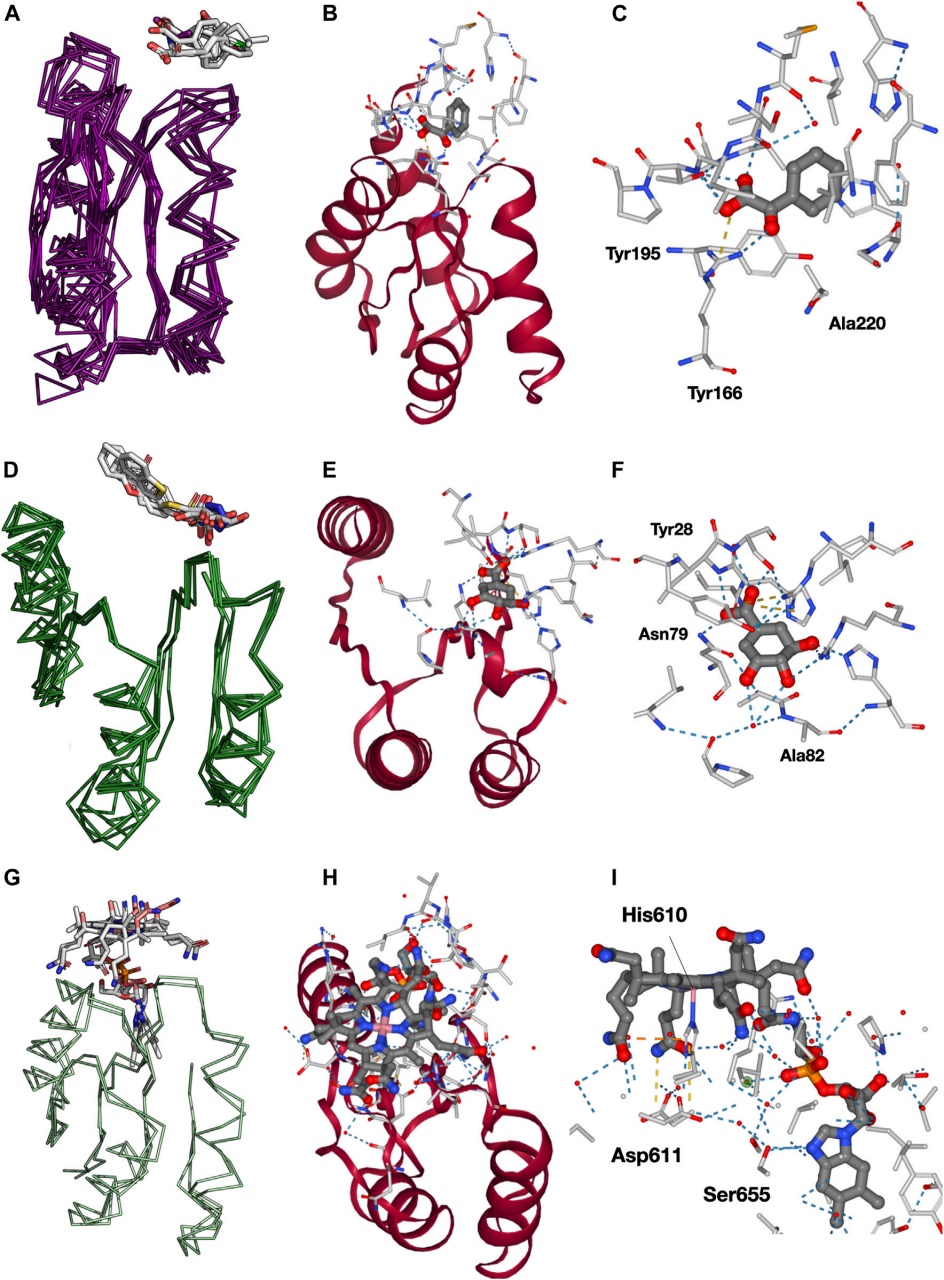
Analysis of binding modes in cluster 13 for superfamily c.93.1, c.23.13, and c.23.6. **(A)** Domains in c.93.1 that contain cluster 13 and bind a ligand. **(B)** Example of tyrosine (Tyr) bound to domain d3snra_ and its interactions with cluster 13 (red). **(C)** Closer view onto the binding pocket (https://fuzzle.uni-bayreuth.de/2.0/ligands/detailed/d3td9a_/135/219/. **(D)** Domains in c.23.13 that contain cluster 13 and bind a ligand. **(E)** 3-dehydroshikimate (DHK) bound to domain d1gtza_ and its interactions with cluster 13 (red). **(F)** Closer view on the binding pocket (https://fuzzle.uni-bayreuth.de/2.0/ligands/detailed/d1gtza_/6/81). **(G)** The two domains in c.23.6 that contain cluster 13 and bind a ligand. **(H)** Cobalamin (B12) bound to domain d1reqa2_ and its interactions with cluster 13 (red). **(I)** Closer view onto the binding pocket. Most of B12 interactions are performed within the fragment (https://fuzzle.uni-bayreuth.de/2.0/ligands/detailed/d1reqa2/598/687).

To this end, we downloaded the PyMOL session with a superposition of all cluster 13-containing domains, available from the domain-centered network view for all fragments (Figure 3, middle). Despite their large sequence divergence, the backbone of the structures is quite conserved (Supplementary Figure S2). In the superposition, we observe several ligands bound to the fragment, mostly at the top position, and some other ligands and solvents interacting with different regions of the protein. We noticed that several of these ligands correspond to additives commonly found in crystallization media, which were discarded from our analysis (see Methods). Ligands within 4 Å of any heavy atom of cluster 13 are summarized in Table 1. Not all superfamilies containing cluster 13 are shown, since from the 12 superfamilies only 8 have ligands bound, and only 4 have 2 or more representatives.

**TABLE 1:** List of domains containing cluster 13 with bound ligands.

Domain	PDB code	Superfamily	Compound name
d2obxa_	INI	c.16.1	5-nitro-6-ribityl-amino-2,4 (1 h,3 h)-pyrimidinedione
d1gtza_	DHK	c.23.13	3-dehydroshikimate
d2y71a_	CB6	c.23.13	(1r,4s,5r)-1,4,5-trihydroxy-3-[(5-methyl-1-benzothiophen-2-yl)methoxy]cyclohex-2-ene-1-carboxylic acid
d2c4wa_	GAJ	c.23.13	N-tetrazol-5-yl 9-oxo-9h-xanthene-2 sulphonamide
d5ydba_	DQA	c.23.13	3-dehydroquinic acid
d2xdaa_	JPS	c.23.13	(4r,6r,7s)-2-(2-cyclopropylethyl)-4,6,7-trihydroxy-4,5,6,7-tetrahydro-1-benzothiophene-4-carboxylic acid
d1a9xb2	CYG	c.23.16	2-amino-4-(amino-3-oxo-propylsulfanylcarbonyl)-butyric acid
d1reqa2	B12	c.23.6	Cobalamin
d1ccwa_	CNC	c.23.6	Co-cyanocobalamin
d2atea_	NIA	c.23.8	4-nitro-5-aminoimidazole ribonucleotide
d2bgga2	U	c.44.3	Uridine-5'-monophosphate
d2x5oa1	VSV	c.5.1	N-({3-[({4-[(z)-(2,4-dioxo-1,3-thiazolidin-5-ylidene)methyl]phenyl}amino)methyl]phenyl}carbonyl)-d-glutamic acid
d1p3da1	UMA	c.5.1	Uridine-5'-diphosphate-n-acetylmuramoyl-l-alanine
d3t23a_	TYR	c.93.1	Tyrosine
d3td9a_	PHE	c.93.1	Phenylalanine
d4nqra_	ALA	c.93.1	Alanine
d3sg0a_	173	c.93.1	Benzoyl-formic acid
d4q6ba_	LEU	c.93.1	Leucine
d3ipca1	LEU	c.93.1	Leucine
d4n0qa_	LEU	c.93.1	Leucine
d3snra_	TYR	c.93.1	Tyrosine

Naturally, the most abundant superfamily is the one of the ribose binding proteins itself (c.93.1, Figure 3A), with 8 domains (Table 1, Supplementary Figure S3). Visualization of these domains reveals that they mostly bind amino acid ligands in a conserved binding mode (Figure 3B), along with benzoyl-formic acid (PDB ligand code 173). As in most PBPs, all ligands in this set bind in the cleft defined by the two protein lobes. Because cluster 13 is located in the N-terminal lobe, it only interacts *via* a few residues with the respective ligand. Two of these interactions are particularly conserved among the sequences, a Tyr or Phe residue, and a Ser, Tyr, or Ala residue (Supplementary Figure S3). We particularly looked at the interactions with Fuzzle 2.0’s detailed viewer for domain d3sg0a_ (https://fuzzle.uni-bayreuth.de/2.0/ligands/detailed/d3sg0a_/139/236/), which contains overall 3 interactions with the fragment: the conserved residues Tyr168 and Ala220 (Supplementary Figure S3) that interact *via* hydrophobic packing and Arg195 that forms a salt bridge with the ligand (Figure 3C). Particularly important is the salt bridge formed between Arg195's guanidium group and ligand 173's carboxyl, an interaction that also appears in two other domains, namely d3snra_ and d3t23a_ (note, that the corresponding PDB entries have been superseded by 3UK0 and 3UK1, respectively).

The second most abundant superfamily is the Type II 3-dehydroquinate dehydratase superfamily (c.23.13), including 5 ligand-binding domains (Supplementary Figure S3). These domains bind ligands that are artificial drugs used as antimicrobial agents (Figure 3D). Figure 3E shows the ligand DHK (3-dehydroshikimate) binding domain d1gtza_ interacting with additional residues outside the fragment. However, two critical interactions are contained in the fragment (Ala82 and Asn79). These interactions are highly conserved among all c.23.13 sequences (Supplementary Figure S3).

The cobalamin (vitamin B_12_)-binding superfamily (c.23.6) is represented by two domains in the receptor set (Supplementary Figure S3). Both domains bind cobalamin variants (cobalamin and co-cyanocobalamin) in a conserved fashion (Figure 3G). Figure 3H shows domain d1reqa2 in complex with B_12_ (cobalamin). The ligand performs most of its interactions with the fragment (Figure 3I), especially with the loop between β1 and α1 with the cobalt-coordinating His610. Other important residues are Asp611 (loop1), and Ser655 (β2; https://fuzzle.uni-bayreuth.de/2.0/ligands/detailed/d1reqa2/598/687).

Other less abundant superfamilies binding ligands are c.5.1, c.16.1, c.44.3, and c.23.16, shown in Supplementary Figure S4. Interestingly, superfamily c.5.1 uses a different mode of binding, with the ligand bound between helices. A superposition with domain d2fn9a_ reveals a different topology where the β-sheets do not exactly superimpose, leaving β3 for d2fn9a_ unmatched. Superfamily c.23.16 contains only one domain with chemical entities, which however only interact with residues mostly outside cluster 13 of domain d1a9xb2 (Supplementary Figure S4B). Superfamily c.44.3 with its representative d2bgga2 binds two molecules of uridine-5'-monophosphate (Supplementary Figure S4C). The ligand binds at the edge of d2bgga2’s β4, which also corresponds to the terminal part of the domain. Superfamily c.16.1 is represented by domain d2obxa_, which binds ligand 5-nitro-6-ribityl-amino-2,4 (1 h, 3 h)-pyrimidinedione (NRP, PDB ligand code INI). All interactions for this ligand are contained in the fragment. These examples show that protein ligand interactions often occur at similar positions in a protein corresponding to the fragments detected in Fuzzle. However, the mode of binding of various ligands in different homologous proteins may vary, e.g. as described for the superfamily c.5.1.

## Discussion

We recently published the Fuzzle (Fold Puzzle) database which contains a set of evolutionarily related protein fragments that can also be used for protein design. It is known that modern proteins evolved by replicating and recombining smaller sequence fragments. Fuzzle offers the opportunity to identify these fragments and to mimic evolutionary processes in the lab as well as to build new proteins. In this updated version of Fuzzle 2.0, we enhanced the analysis tools and extended them to include detailed information about protein fragment-ligand interactions. This extension now enables the identification and analysis of ligands and their interactions with a conserved fragment. As a note of caution: since Fuzzle is based on a non-redundant dataset of SCOPe, some ligand information might be incomplete. Thus, it will be still necessary to consult the literature or other databases for an in-depth analysis of a specific protein-ligand interaction.

Using a periplasmic binding protein (PBP) fragment as an example, we demonstrated the new features of Fuzzle 2.0. SCOP places this fold into a single superfamily, periplasmic binding protein-like I (PBP-like I superfamily, c.93.1). Its fold consists of two similar intertwined lobes with 3 layers (α/β/α), each composed of a parallel six-stranded β-sheet with the order 213456 (Figure 1A). There also exists a two-lobed PBP-like II fold (c.94), with a somewhat different topology. The two lobes in both folds define a small hinge region that recognizes a large number of ligands and ions in bacteria. PBPs exist in an open and closed conformation, with the open conformation predominating in the absence of ligands (Kröger et al., 2021). Such conformational plasticity have led to PBPs being widely used in biosensing applications (Grünewald, 2014). Based on structural consideration alone it has long been proposed that the PBP-like I fold arose *via* gene duplication from a flavodoxin-like fold (c.23) (Louie, 1993; Fukami-Kobayashi et al., 1999). In fact, a protein chimera could be built through combination of fragments from these two folds (PDB id: 4QWV). A similar postulated duplication event has also recently been explored for the emergence of the two-lobed HemD-like fold from flavodoxin-like proteins (Toledo-Patiño et al., 2019), combining sequence and structural analysis with experimental reconstruction.

Here, we have focused our analysis on the ribose binding protein from *Thermotoga maritima* (tmRBP), a single domain protein (d2fn9a_). The domain contains many sequence similarities to other superfamilies, especially in its N-terminal region (Figure 1B). This region corresponds to a conserved fragment spanning 3 helices and four β-strands (Figure 2). The fragment occurs in 63 domains of 12 different superfamilies, and thus offers a great prospect for protein engineering. A detailed protein-ligand analysis was described for 22 of the domains, distributed over seven of the 12 identified superfamilies (Table 1).

The dataset of ligand-binding domains offers opportunities for protein design. While the engineering of ligand-binding pockets has become more successful over the years, it is still difficult. Now, reusing ready-made parts from existing proteins can help overcome some of the difficulties. Therefore, we suggest chimeragenesis by replacement in which the corresponding fragment in d2fn9a_ gets replaced by a homologous fragment binding a ligand such as the one in INI-binding d2obxa_ domain. Such an approach has been successfully applied in several instances and offers a novel route for functional diversification (Lechner et al., 2018). Another interesting opportunity that this approach offers is to test evolution by protein engineering as was previously shown for the HemD fold, another bilobular protein. The protein could be dissected into its two lobes, one of which was shown to fold by itself into the related flavodoxin-like fold c.23 (Toledo-Patiño et al., 2019). We would expect similar behaviour for the PBP-like folds.

One question that remains is whether the lower PBP-lobe could adopt the functionality of some of its related proteins like those described above belonging to the flavodoxin-like fold. Here, we have shown that domains of several superfamilies (c.93.1, c.23.6, and c.5.1) bind different ligands at similar regions in the protein structure; however, the mode of binding can differ. This region represents a conserved fragment and therefore strengthens the hypothesis that domains contain conserved building blocks even shared by seemingly unrelated folds. This observation gives rise to the possibility to identify potential ligands that could bind to a domain. In the example described the analysis suggests that the identified fragment in d2fn9a_ could be capable of recognizing ligands B12 or INI after performing several rounds of mutations either by protein engineering or directed evolution.

Overall, we believe that the new version of Fuzzle will be a valuable tool for various fields of research. On the one hand Fuzzle 2.0 allows evolutionary biologists to strengthen the evidence for common ancestry and on the other hand allows protein designers to use this information in transferring ligand binding sites into other protein scaffolds.

## Data Availability

Publicly available datasets were analyzed in this study. This data can be found here: https://fuzzle.uni-bayreuth.de/2.0.

## References

[B1] Alva V. Söding J. Lupas A. N. (2015). A Vocabulary of Ancient Peptides at the Origin of Folded Proteins. Elife 4, e09410. 10.7554/eLife.09410.001 26653858 PMC4739770

[B2] Alva V. Lupas A. N. (2018). From Ancestral Peptides to Designed Proteins. Curr. Opin. Struct. Biol. 48, 103–109. 10.1016/j.sbi.2017.11.006 29195087

[B3] Bharat T. A. M. Eisenbeis S. Zeth K. Höcker B. (2008). A -barrel Built by the Combination of Fragments from Different Folds. Proc. Natl. Acad. Sci. 105, 9942–9947. 10.1073/pnas.0802202105 18632584 PMC2481348

[B4] Cuneo M. J. Beese L. S. Hellinga H. W. (2008). Ligand-induced Conformational Changes in a Thermophilic Ribose-Binding Protein. BMC Struct. Biol. 8, 50. 10.1186/1472-6807-8-50 19019243 PMC2630998

[B5] Farías-Rico J. A. Schmidt S. Höcker B. (2014). Evolutionary Relationship of Two Ancient Protein Superfolds. Nat. Chem. Biol. 10, 710–715. 10.1038/nchembio.1579 25038785

[B6] Ferruz N. Lobos F. Lemm D. Toledo-Patino S. Farías-Rico J. A. Schmidt S. (2020). Identification and Analysis of Natural Building Blocks for Evolution-Guided Fragment-Based Protein Design. J. Mol. Biol. 432, 3898–3914. 10.1016/j.jmb.2020.04.013 32330481 PMC7322520

[B7] Ferruz N. Noske J. Höcker B. (2021). Protlego: a Python Package for the Analysis and Design of Chimeric Proteins. Bioinformatics, btab253. 10.1093/BIOINFORMATICS/BTAB253 33901273 PMC8504633

[B30] Fox K. N. Brenner E. S. Chandonia J. M. (2014). SCOPe: Structural Classification of Proteins—Extended, Integrating SCOP and ASTRAL Data and Classification of New Structures. Nucleic Acids Res. 42, D304–D309. 10.1093/nar/gkt1240 24304899 PMC3965108

[B8] Fukami-Kobayashi K. Tateno Y. Nishikawa K. (1999). Domain Dislocation: A Change of Core Structure in Periplasmic Binding Proteins in Their Evolutionary History. J. Mol. Biol. 286, 279–290. 10.1006/jmbi.1998.2454 9931266

[B9] Godden J. W. Xue L. Bajorath J. (2000). Combinatorial Preferences Affect Molecular Similarity/Diversity Calculations Using Binary Fingerprints and Tanimoto Coefficients. J. Chem. Inf. Comput. Sci. 40, 163–166. 10.1021/ci990316u 10661563

[B31] Goncearenco A. Berezovsky I. N. (2015). Protein Function From Its Emergence to Diversity in Contemporary Proteins. Phys. Biol. 12, 045002. 10.1088/1478-3975/12/4/045002 26057563

[B10] Grünewald F. S. (2013). Periplasmic Binding Proteins in Biosensing Applications. Bioanal. Rev. 1, 205–235. 10.1007/11663_2013_7

[B11] Horowitz N. H. (1945). On the Evolution of Biochemical Syntheses. Proc. Natl. Acad. Sci. 31, 153–157. 10.1073/pnas.31.6.153 16578152 PMC1078786

[B12] Jensen R. A. (1976). Enzyme Recruitment in Evolution of New Function. Annu. Rev. Microbiol. 30, 409–425. 10.1146/annurev.mi.30.100176.002205 791073

[B13] Kröger P. Shanmugaratnam S. Ferruz N. Schweimer K. Höcker B. (2021). A Comprehensive Binding Study Illustrates Ligand Recognition in the Periplasmic Binding Protein PotF. Structure 29, 433–443. 10.1016/j.str.2020.12.005 33406388

[B14] Laurino P. Tóth-Petróczy Á. Meana-Pañeda R. Lin W. Truhlar D. G. Tawfik D. S. (2016). An Ancient Fingerprint Indicates the Common Ancestry of Rossmann-fold Enzymes Utilizing Different Ribose-Based Cofactors. PLOS Biol. 14, e1002396. 10.1371/journal.pbio.1002396 26938925 PMC4777477

[B15] Lechner H. Ferruz N. Höcker B. (2018). Strategies for Designing Non-natural Enzymes and Binders. Curr. Opin. Chem. Biol. 47, 67–76. 10.1016/j.cbpa.2018.07.022 30248579

[B16] Longo L. M. Jabłońska J. Vyas P. Kanade M. Kolodny R. Ben-Tal N. (2020). On the Emergence of P-Loop Ntpase and Rossmann Enzymes from a Beta-Alpha-Beta Ancestral Fragment. Elife 9, 1–16. 10.7554/ELIFE.64415 PMC7758060 33295875

[B17] Louie G. V. (1993). Porphobilinogen Deaminase and its Structural Similarity to the Bidomain Binding Proteins. Curr. Opin. Struct. Biol. 3, 401–408. 10.1016/S0959-440X(05)80113-7

[B18] Narunsky A. Kessel A. Solan R. Alva V. Kolodny R. Ben-Tal N. (2020). On the Evolution of Protein-Adenine Binding. Proc. Natl. Acad. Sci. USA 117, 4701–4709. 10.1073/pnas.1911349117 32079721 PMC7060716

[B19] Nepomnyachiy S. Ben-Tal N. Kolodny R. (2017). Complex Evolutionary Footprints Revealed in an Analysis of Reused Protein Segments of Diverse Lengths. Proc. Natl. Acad. Sci. USA 114, 11703–11708. 10.1073/pnas.1707642114 29078314 PMC5676897

[B20] Ohta T. (2000). Mechanisms of Molecular Evolution. Phil. Trans. R. Soc. Lond. B 355, 1623–1626. 10.1098/rstb.2000.0724 11127908 PMC1692885

[B22] Romero Romero M. L. Yang F. Lin Y.-R. Toth-Petroczy A. Berezovsky I. N. Goncearenco A. (2018). Simple yet Functional Phosphate-Loop Proteins. Proc. Natl. Acad. Sci. USA 115, E11943–E11950. 10.1073/pnas.1812400115 30504143 PMC6304952

[B23] Romero-Romero S. Kordes S. Michel F. Höcker B. (2021). Evolution, Folding, and Design of TIM Barrels and Related Proteins. Curr. Opin. Struct. Biol. 68, 94–104. 10.1016/j.sbi.2020.12.007 33453500 PMC8250049

[B24] Rose A. S. Bradley A. R. Valasatava Y. Duarte J. M. Prlić A. Rose P. W. (2018). NGL Viewer: Web-Based Molecular Graphics for Large Complexes. Bioinformatics 34, 3755–3758. 10.1093/bioinformatics/bty419 29850778 PMC6198858

[B25] Schmidt S. Dandekar T. (2002). Gene Regulations and Metabolism - Postgenomic Computational Approaches. Editor Collado-Vides J. Hofestädt R. (MIT Press Cambridge, Massachusetts London, England).

[B26] Sikosek T. Chan H. S. (2014). Biophysics of Protein Evolution and Evolutionary Protein Biophysics. J. R. Soc. Interf. 11, 20140419. 10.1098/rsif.2014.0419 PMC4191086 25165599

[B32] Söding J. (2005). Protein Homology Detection by HMM-HMM Comparison. Bioinformatics 21(7), 951–960. 10.1093/bioinformatics/bti125 15531603

[B27] Toledo-Patiño S. Chaubey M. Coles M. Höcker B. (2019). Reconstructing the Remote Origins of a Fold Singleton from a Flavodoxin-like Ancestor. Biochemistry 58, 4790–4793. 10.1021/acs.biochem.9b00900 31724394 PMC6968885

[B28] Weininger D. (1988). SMILES, a Chemical Language and Information System. 1. Introduction to Methodology and Encoding Rules. J. Chem. Inf. Model. 28, 31–36. 10.1021/ci00057a005

[B29] Yang J. Roy A. Zhang Y. (2013). BioLiP: A Semi-manually Curated Database for Biologically Relevant Ligand-Protein Interactions. Nucleic Acids Res. 41, D1096–D1103. 10.1093/nar/gks966 23087378 PMC3531193

[B33] Zhang Y. Skolnick J. (2005). TM-Align: A Protein Structure Alignment Algorithm Based on the TM-Score. Nucleic Acids Res. 33(7), 2302–2309. 10.1093/nar/gki524 15849316 PMC1084323

