# Supplementary material for: Fuzzle 2.0: Ligand Binding in Natural Protein Building Blocks
Source: Front Mol Biosci. 2021 Aug 18;8:715972. doi: 10.3389/fmolb.2021.715972 (PMC8416435; doi:10.3389/fmolb.2021.715972)

# Supporting Information for:

**Fuzzle 2.0: Ligand Binding in Natural Protein Building Blocks**

Noelia Ferruz[1*], Florian Michel[1], Francisco Lobos[1], Steffen Schmidt[2], Birte Höcker[1*]
[1]Department of Biochemistry, University of Bayreuth, 95447 Bayreuth, Germany,
[2]Computational Biochemistry, University of Bayreuth, 95447 Bayreuth, Germany.
*** Correspondence:**
Corresponding Author
birte.hoecker@uni-bayreuth.de, noelia.ferruz-capapey@uni-bayreuth.de
Keywords: web server, protein evolution, protein design, protein fragment, flavodoxin-like fold, periplasmic binding protein.

**Table S1: Statistics of Datasets.** Fuzzle 2.0 allows to access two datasets, SCOP 2.06 and SCOP 2.07. The last row reports the pairwise hits using the filtering criteria reported in the manuscript (HHSearch Probability > 70, TM-score > 0.3 with at least 10 $C_\alpha$-Atoms superposed, a RMSD below 3.0 Å, and a ratio between the sequence and structure lengths of maximum 1.25.

| Dataset | SCOP 2.06 | SCOP 2.07 |
|---|---|---|
| Families | 4,783 | 4,849 |
| Superfamilies | 2,006 | 2,024 |
| Folds | 1,221 | 1,232 |
| Fuzzle hits | 8,109,195 | 10,434,359 |
| Fuzzle hits (filtered) | 4,970,087 | 6,255,666 |

**Table S2:** The 18 **clusters found in the ribose binding protein**. The cluster identifiers correspond to Figure 3. The start/end positions match the amino acid sequence of d2fn9a_. The number of domains that are contained in each cluster is shown in the last column. Note, that multiple fragments can be found within a single domain but are sorted into different clusters, e.g. 4 domains are found in both cluster 51 and 13 and therefore are counted twice, resulting in a greater number of total reported domains.

| Cluster | Start | End | Domains |
|---|---|---|---|
| 0 | 2 | 280 | 49 |
| 2 | 2 | 223 | 2 |
| 6 | 5 | 135 | 2 |
| 8 | 2 | 123 | 7 |
| 9 | 129 | 264 | 7 |
| 11 | 112 | 252 | 2 |
| 13 | 11 | 87 | 63 |
| 18 | 47 | 115 | 5 |
| 19 | 2 | 116 | 2 |
| 21 | 2 | 101 | 3 |
| 23 | 169 | 256 | 3 |
| 25 | 44 | 87 | 5 |
| 26 | 17 | 106 | 7 |
| 29 | 149 | 234 | 2 |
| 30 | 53 | 105 | 2 |
| 51 | 15 | 93 | 5 |
| 73 | 152 | 257 | 3 |
| 103 | 22 | 120 | 2 |

**Figure S1: Most common ligands found in conserved fragments.**

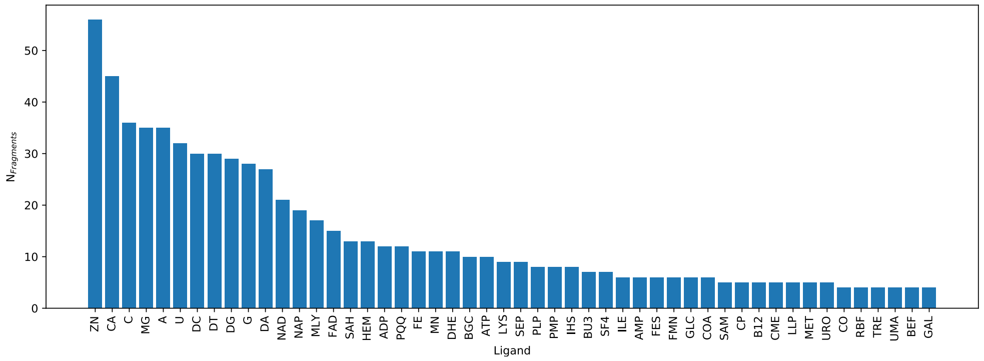


**Figure S2: Superposition of all domains that contain cluster 13** (https://fuzzle.uni-bayreuth.de/2.0/super/pymol/cluster/d2fn9a__13/70/3.0/1.25).

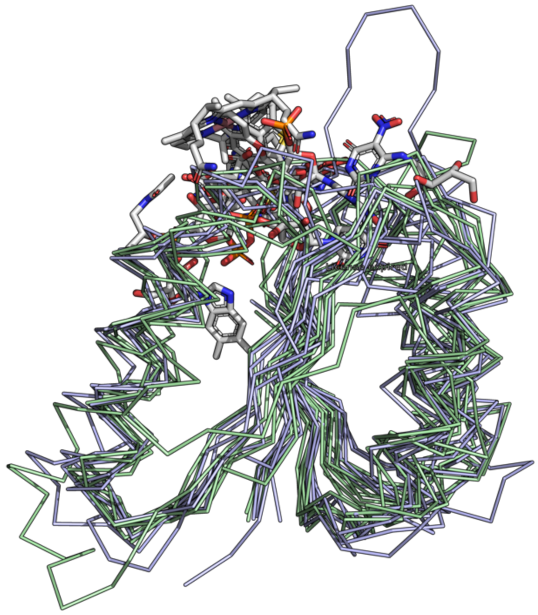

**Figure S3: Sequence alignment for the ligand-binding domains in superfamilies c.93.1, c.23.13, and c.23.6.** Interactions with the ligands are highlighted.

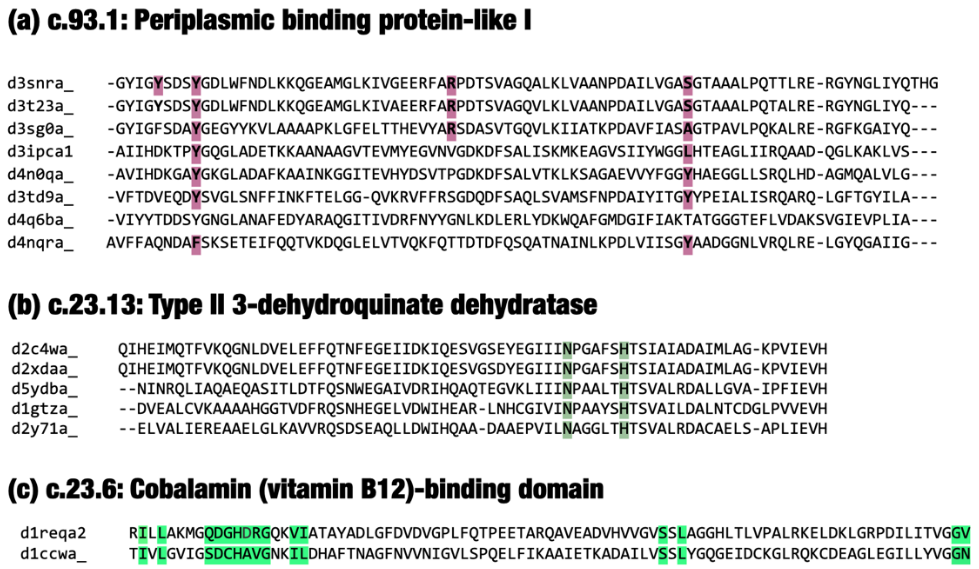


**Figure S4: Ligand-binding domains containing cluster 13 from less numerous superfamilies.**

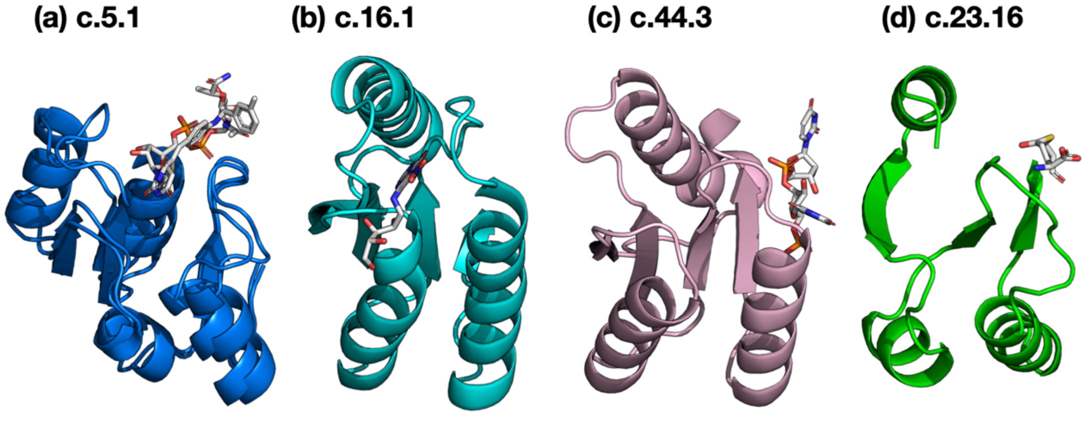

Supplement: Supplementary file 1 [file DataSheet1.pdf]
